# Chemical Recycling of Polyolefins Waste Materials Using Supercritical Water

**DOI:** 10.3390/polym14204415

**Published:** 2022-10-19

**Authors:** Maja Čolnik, Petra Kotnik, Željko Knez, Mojca Škerget

**Affiliations:** 1 Laboratory for Separation Processes and Product Design, Faculty of Chemistry and Chemical Engineering, University of Maribor, Smetanova 17, SI-2000 Maribor, Slovenia; 2 Department for Chemistry, Faculty of Medicine, University of Maribor, Taborska 8, SI-2000 Maribor, Slovenia

**Keywords:** polypropylene, polyolefins, supercritical water, plastics waste, chemical recycling

## Abstract

In the following work, the hydrothermal degradation of polypropylene waste (PP) using supercritical water (SCW) has been studied. The procedure was carried out in a high-pressure, high-temperature batch reactor at 425 °C and 450 °C from 15 to 240 min. The results show a high yield of the oil (up to 95%) and gas (up to 20%) phases. The gained oil phase was composed of alkanes, alkenes, cycloalkanes, aromatic hydrocarbons, and alcohols. Alkanes and alcohols predominated at 425 °C and shorter reaction times, while the content of aromatic hydrocarbons sharply increased at higher temperatures and times. The higher heating values (HHVs) of oil phases were in the range of liquid fuel (diesel, gasoline, crude and fuel oil), and they were between 48 and 42 MJ/kg. The gas phase contained light hydrocarbons (C_1_–C_6_), where propane was the most represented component. The results for PP degradation obtained in the present work were compared to the results of SCW degradation of colored PE waste, and the potential degradation mechanism of polyolefins waste in SCW is proposed. The results allowed to conclude that SCW processing technology represents a promising and eco-friendly tool for the liquefaction of polyolefin (PE and PP) waste into oil with a high conversion rate.

## 1. Introduction

Plastic is a trendy material in all sectors, as it is a useful material that is inexpensive, technically sophisticated, and easy to make [1,2,3]. In 2020, the global plastic production was 367 million metric tons of which 55 million metric tons were produced in Europe [4]. Due to the impacts of COVID-19, global plastic production decreased by approximately 0.3% compared to 2019, but due to the incredible versatility and utility of plastics, the volume of the plastics market is expected to continue to grow in the future [5].

The largest segment of plastics produced is represented by thermoplastics (85%), of which the most common are polyolefins (polyethylene (PE) and polypropylene (PP)) [6]. Today, polyolefins are among the most important and useful commodity plastic materials in the world and are used in many applications as bags, films, containers, toys, industrial wraps and films, pipes, gas pipes, electrical equipment (HDPE and LDPE) [7,8] and flowerpots, cables, pipes, food packaging and medical equipment (PP) [7,9]. These three materials together represent more than 50% of all produced plastic in the world [6]. Market analysis shows that in 2021, PP production reached 76 million metric tons [10], while PE production was significantly higher and amounted to 107 million metric tons worldwide [11]. According to forecasts, the production of polyolefins should grow by another 3.6% until 2029 [10,11].

Plastic waste pollution has become one of the most pressing environmental issues, as the rapidly growing production of plastic products exceeds their recyclability [12]. Besides, the lifetime of plastics is estimated at hundreds and thousands of years [13,14]. The biggest problem is in disposable plastic products, which present 40% of all plastics produced each year [15]. Many products (plastic bags and food packaging) have a lifetime of a few minutes to hours. Immediately after use, they are discarded and accumulate in landfills (including in nature and the seas), where they decompose into microplastics and nanoplastics [16]. In addition, additives (colorants, plasticizers, softeners, lubricants, etc.) [17] and other degradation products are released into the environment where these plastic materials are disposed. Unfortunately, it is a frightening fact that more than one million tons of plastic waste end up in the oceans every year [1,18].

Currently, two main processes are used to recycle plastic waste: incineration and mechanical recycling. Although incineration with energy recovery is sufficiently efficient in disposing of these materials and is capable of processing various plastic wastes simultaneously, it is not preferred technology in the circular economy transition policies because it results in a waste of material resources and it releases lots of harmful and environmental polluting gases. On the other hand, mechanical recycling is not applicable to polymer blends and requires energy consumption for washing and drying. Furthermore, colored plastic waste can only be converted to dark-colored plastic products and the quality of recycled plastic is lower than the original plastic [19]. Recently chemical recycling, which could turn waste plastics into secondary raw materials, has become increasingly important. Hydrothermal processes (HTP) based on sub- and supercritical water (SubCW and SCW) represent a great potential to solve the problems of plastic pollution [20]. The use of SCW shows several advantages compared to other chemical recycling methods of polyolefins (thermal and catalytic degradation) in terms of process performance, economy, and low environmental impact, especially because it is potentially useful for processing technically difficult waste, such as mixed plastics and plastics contaminated with organic waste [3]. The SubCW and SCW method is suitable for converting various wastes generated in households and industries into value-added products (gases, valuable chemicals, fuels) [21]. Due to its low viscosity, high diffusivity, low dielectric constant, and high ionic product, SCW represents a homogeneous highly reactive reaction medium for the decomposition of hydrocarbons with a high reaction rate [22]. In addition, HTP processes also eliminate problem arising from the low thermal conductivity of polymers, manage to deal with common additives in these matrices, such as flame retardants, or stabilizers [20,23] as well as colorants [24].

Investigations dealing with degradation of polyolefins in SCW have grown in recent years [25,26,27,28,29,30]. Several studies on the hydrolysis of virgin PE (HDPE and LDPE) using SCW have been conducted. Degradation of virgin HDPE to oils in SCW was investigated by Su et al. and it was found that conditions which favor the high oil yield (more than 90%) are temperature around 460–480 °C and short reaction time (1–30 min) [26]. Similarly, Zhang et al. investigated the degradation of virgin HDPE to oil in a continuous SCW reactor. It was reported that the highest yield of oil product (79%) was determined at 530 °C, while the yield of the gas increased from 3% to 22% as the temperature increased from 500 °C to 550 °C [27]. In our previous work, where the degradation of virgin LDPE and colored HDPE waste in SCW was investigated, it was found that a long reaction time (240 min) and high reaction temperature (450 °C) produce less oil and more gas products [8]. Similar findings were also obtained in work by Jin et al., where virgin HDPE and HDPE waste (milk jugs, grocery bags) were degraded in SCW at temperatures up to 450 °C and reaction times from 0.5 min to 4 h [31]. The content of hydrocarbons in the oil and gas phase produced during the decomposition of polyolefins depends on reaction conditions. Light hydrocarbons in the range of C_1_-C_6_ are formed in the gas phase, while shorter (C_5_-C_17_) and longer hydrocarbons (≥C_18_) are formed in the oil phase. The chemical compositions of the obtained oil phases was similar to gasoline and diesel fractions. The amount of solid residue after the degradation of PE waste varied from 0.1% to 20% depending on the inorganic additives contained in the individual polymer [8,31]. In addition, the catalytic SCW gasification of polyolefin plastics at 450 °C and 60 min was studied by Onwudili et al. and and methane was reported to be the most abundant gas component in the resulting gas mixture [32].

Furthermore, there are also several investigations dealing with the degradation of virgin PP in SCW at the temperatures from 380 to 500 °C, at different reaction times (up to 6 h), and in different reaction atmospheres (air, N_2_, H_2_, O_2_, and H_2_O_2_) [30,33,34]. Similar to PE degradation, the oil and gas yields from virgin PP increased with increasing reaction temperature and time. In the study by Chen et al. it was found that the optimal reaction conditions to achieve high oil yield (91 wt %) were 425 °C and 4 h of reaction time. At these conditions, an additional SCW test was carried out using a waste container made of PP, where a similar oil yield (90 wt %) was obtained. The oil products from virgin PP contained various olefins, paraffins, alicyclic and aromatic compounds. About 90% of the oil components had the same boiling point range as petroleum naphtha (25–200 °C). The main hydrocarbons in the gas mixture were C_3_, C_4_, and C_5_ [30]. In the next study, Bai et al. investigated the gasification of PP waste in SCW in the quartz tube reactor at high temperatures (500–800 °C) and reaction time from 2 to 60 min. It was found that the suitable reaction conditions for gasification of PP waste are 750 °C for 60 min, and 79.86 wt % of carbon conversion is obtained with addition of formic acid [35].

All these studies show an excellent contribution to understanding the degradation of virgin polyolefins (PE and PP) in SCW, however according to above reviewed literature, almost no studies on the degradation of the real colored or colorless PP waste in SCW have been published yet and there are no data on the chemical composition of the oil and gas phases obtained from PP waste.

The main goal of the present research is to investigate the chemical recycling of real colorless and colored PP waste by SCW at temperatures from 425 °C to 450 °C for reaction times from 15 min to 240 min and to determine the optimal reaction conditions for obtaining the gas and oil phases in high yields and of specified composition. A major motivation of this research is also to compare the composition of the resulting oil and gas phases obtained from PP and PE waste, in order to verify whether it would be possible to simplify the process of sorting of plastic waste. Therefore, a comparison of the results obtained for PP with the results of hydrothermal degradation of PE from our previous work [8] has also been done, the theoretical HHVs of oil phases of both polymers were estimated and the degradation mechanism of polyolefins in SCW was proposed.

## 2. Materials and Methods

### 2.1. Materials

Colorless and colored PP waste (mixed PP waste of different colors (black and blue)) was kindly donated from Surovina Maribor, Slovenia. Dichloromethane (≥99.8%) and Potassium hydrogen phthalate (99.95%) were supplied by Sigma-Aldrich. Nitrogen (99.5%) and helium (99.999%) were supplied by Messer (Ruše, Slovenia).

### 2.2. Characterization of Initial Material

Ultimate analysis of PP waste (~ 1.5–2.5 mg) was carried out with CHNS/O elemental analyzer (Perkin Elmer 2400 Series II System Analyzer) at 975 °C under oxygen atmosphere. The content of oxygen in waste PP material was calculated by the equation: O (wt %) = 100 − (C + H + N + S) (wt %) [36].

The thermal properties of PP waste were performed by Mettler Toledo differential scanning calorimetric (DSC) (TGA/DSC1 STAR) system. The small amount of sample (20 ± 5 mg) was weighed into the aluminium pan with lid and analysed. First, each sample was heated from 20 °C to 200 °C to obtain a molten phase, then cooled to 20 °C to achieve crystallization, and then again heated to 200 °C to achieve melting. Crystallization temperature (*T*_c_) and crystallization enthalpy (Δ*H*_c_) were determined from the cooling step, while melting temperature (*T*_m_) and melting enthalpy (Δ*H*_m_) were determined from the second heating sequence. All experiments were performed in triplicate at a constant nitrogen flow of 20 mL/min and at a constant heating and cooling rate of 10 K/min.

### 2.3. Supercritical Water Treatment

**Figure 1:**
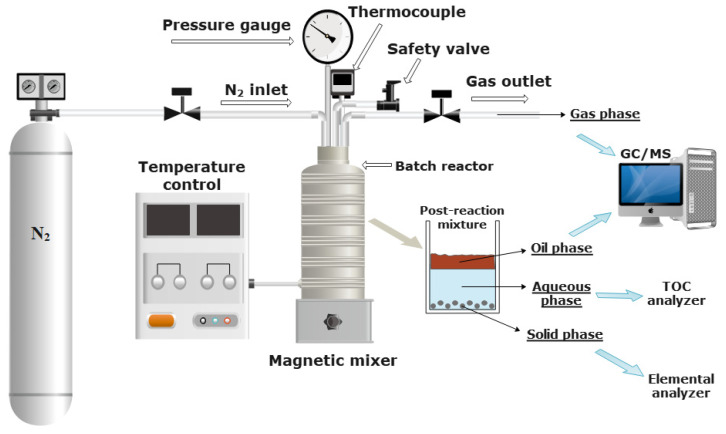
High pressure/temperature batch reactor system for chemical recycling of colorless and colored PP waste in SCW.

The SCW degradations of colorless and colored PP waste were carried out in a batch reactor (series 4740 Stainless Steel, Parr Instruments, Moline, IL, USA) with a volume of 75 mL, which can operate at high temperatures and high pressures up to 540 °C and 580 bar, respectively [8] (Figure 1). The operating conditions investigated were temperatures of 425 °C and 450 °C, reaction time from 15–240 min, and water/PP ratio of 5 mL/1 g. Colored or colorless PP waste and water mixtures were introduced into the reactor. Before treatment, the reactor was first purged with N_2_ to remove the residual air and then filled with N_2_ to set the initial pressure to 5 bar. Then, the reactor was heated with an electric wire to the designated temperature. The heating times to 425 °C and 450 °C were 34 and 39 min, respectively. The temperature was kept constant for the desired reaction time (15–240 min), and the reaction mixture was stirred at 800 rpm^−1^. The pressure at the constant temperature during the reaction was between 290 and 400 bar. After SCW degradation of PP waste, the reactor was rapidly cooled in an ice bath. When the temperature in the reactor reached room temperature, the generated gases were carefully collected into sampling Tedlar bags through a valve and then directly analyzed by gas chromatography with mass spectrometry (GC-MS). The total volume of generated gas was measured with a disposal of water in graduated cylinder and then, the yield of gas phase was determined based on ideal gas law. Afterwards, the reactor was opened, the post-reaction mixture was transferred into a glass beaker and separated into oil and aqueous phase by dichloromethane (CH_2_Cl_2_). Obtained oil-CH_2_Cl_2_ mixture was first filtered to remove the solid residue. The solid residue was washed, dried, and weighed. The oil-CH_2_Cl_2_ mixture was evaporated in a rotary evaporator under atmospheric pressure to remove CH_2_Cl_2_ and weighted. The yield of oil, solid residue and gas phase were calculated by the following Formulas (1)–(3) [8]:

(1) $$\gamma_{\mathrm{oil}}=\frac{m_{\mathrm{oil}}}{m_{\mathrm{PP}\;\mathrm{waste}}}\times 100\%$$

(2) $$\gamma_{\mathrm{SP}}=\frac{m_{\mathrm{SP}}}{m_{\mathrm{PP}\;\mathrm{waste}}}\times 100\%$$

(3) $$\gamma_{\mathrm{unreacted}\;\mathrm{PP}\;\mathrm{waste}}=\frac{m_{\mathrm{unreacted}\;\mathrm{PP}\;\mathrm{waste}}}{m_{\mathrm{PP}\;\mathrm{waste}}}\times 100\%$$

where, $\gamma_{\mathrm{oil}}$, $\gamma_{\mathrm{SP}\;}$ and $\gamma_{\mathrm{unreacted}\;\mathrm{PP}\;\mathrm{waste}}$ present yields of oil, solid, and non-degraded PP waste, respectively. $m_{\mathrm{oil}}$, $m_{\mathrm{SP}}$ and $m_{\mathrm{unreacted}\;\mathrm{PP}\;\mathrm{waste}}$ are weights of oil, solid, and unreacted PP waste obtained by SCW degradation of PP plastics. $m_{\mathrm{PP}\;\mathrm{waste}}$ is the mass of colored or colorless PP waste loaded in the reactor.

### 2.4. GC-MS Analysis of Oil and Gas Products

The chemical composition of gas and oil products obtained by hydrothermal degradation of PP waste were analysed using a GC-MS system consisted of Shimadzu GC-2010 gas chromatograph and Shimadzu GC-MS 2010 QC Ultra mass selective detector.

The volatile oily compounds were separated on an HP-1 (dimensions 30 m × 0.25 mm × 0.25 µm) column with helium as a carrier gas at a 1 mL/min flow rate. Samples were injected in splitless mode and the temperature of the injector was 250 °C. The oven temperature was initially 40 °C. The separation was achieved using a temperature gradient: 40 °C for 1 min, then increased at 10 °C/min to temperature 180 °C, and at 5 °C/min to final temperature 230 °C and held it for 5 min [8].

The gas compounds were separated on HP PLOT/Q (dimensions 30 m × 0.32 mm × 20 µm) column with helium as a carrier gas at a flow rate of 0.87 mL/min. Samples were injected in split mode at ratio 1:100 at the temperature of the injector 250 °C. The oven temperature was initially 35 °C. The separation was achieved using a temperature gradient: an initial temperature 35 °C was held constant for 5 min, then increased at 10 °C/min to a temperature 80 °C, held for 1 min and increased to a final temperature 200 °C at 5 °C/min and held it for 5 min [22].

The mass detector was operated in electron-impact (EI) mode (electron energy 70 mV) at 250 °C with an interface 250 °C. The full-scan mass spectra from 35–500 *m/z* was recorded for analyte identification. All compounds were identified using the NIST library. 

### 2.5. Theoretical Determination of Calorific Value in Oil Phase after SCW of Polyolefins Waste

The higher heating value (HHV) of the oil phase obtained from polyolefins waste was calculated by the Dulong Formula (4) [36].

(4) $$\mathrm{HHV}=\left[8080\cdot C\;+34500\cdot \left(H-\frac{O}{8}\right)\right]\;\mathrm{in}\;\mathrm{kcal}/\mathrm{kg}$$

where C, H, and O are the % of the corresponding elements in the oil phase.

### 2.6. Determination of Total Carbon in the Aqueous Phase

Shimadzu TOC analyzer was used to determine total carbon in aqueous phases. The calibration curve of potassium hydrogen phthalate with concentration ranges up to 1000 ppm was used to calculate total carbon concentration in the aqueous phase after PP waste degradation in SCW. Based on the mass balance, the mass fraction of carbon that was transferred to the aqueous phase during the decomposition of PP waste was estimated.

## 3. Results

### 3.1. Properties of PP Waste

**Figure 2:**
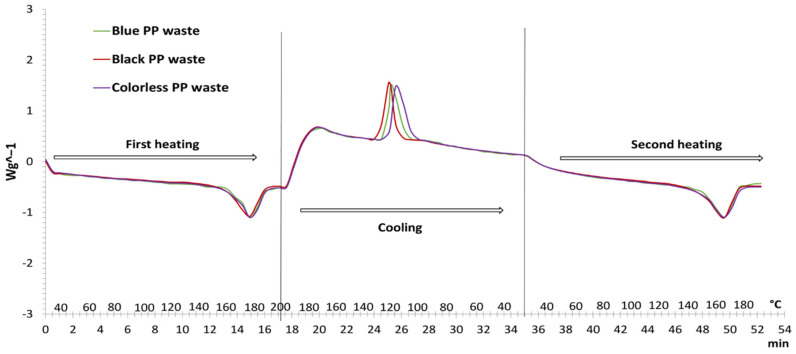
DSC thermograms of melting and crystallization curve of colored and colorless PP waste.

The DCS thermograms of the melting and crystallization curves of colored (black and blue) and colorless PP waste are shown in Figure 2. The obtained thermal properties data (crystallization temperature (*T*_cr_), crystallization enthalpy (Δ*H*_c_), melting temperature (*T*_m_), melting enthalpy (Δ*H*_m_)) and elemental analysis of PP waste are shown in Table 1. The first heating measurement on DSC is performed to remove the thermal history of the sample, which may be due to the processing conditions induced during the preparation of the individual plastics product [37,38]. In general, it is often very useful to measure the cooling curve of the sample (crystallization) and then record the second heating (melting). These additional measurements provide more information of the behavior of the material and gave more important results, as they reflect samples with the same thermal history [38,39].

The DSC results show that the melting point of colorless PP waste (purple line) is at 169.90 °C and it is higher than melting points of the colored PP waste (red and green lines) (Table 1). Moreover, the colorless PP waste has a slightly lower crystallization temperature (119.36 °C) than the colored PP waste during the cooling process. Pedrazolli et al. [38] also reported that incorporation of the filler into PP polymer matrix produces a moderate increase of the crystallization temperature. Similarly, Boparai et al. [37] observed a slight decrease in melting temperature with the reinforcement of nanoparticles in the Nylon 6 matrix. According to the literature and our results, it can be concluded, that the slightly lower melting temperature and a bit higher crystallization temperature indicate that colored PP waste contains more additives and colorants than colorless PP waste. It is similar in the case of elemental analysis, where all colored samples have a higher oxygen content and a lower carbon content than the colorless sample. 

### 3.2. SCW Products Distribution

After SCW degradation of colorless and colored PP waste, four phases (oil, gas, solid, and aqueous) were formed.

#### 3.2.1. Oil Phase

**Figure 3:**
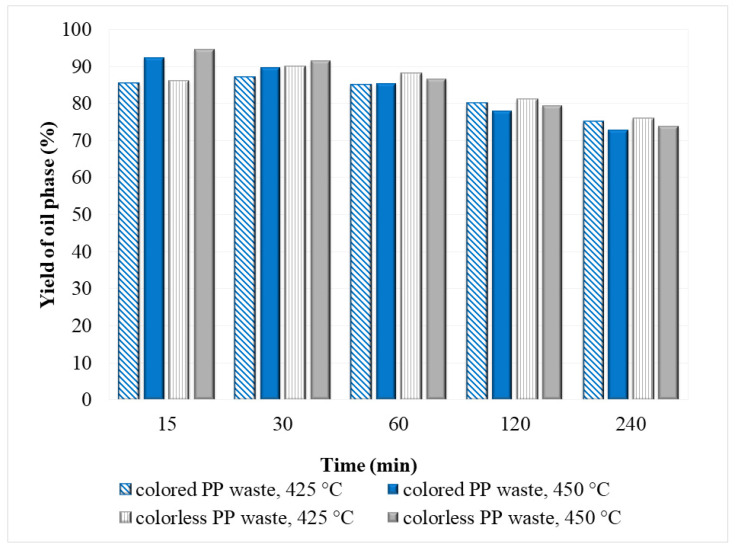
The yield of oil phase after SCW degradation of PP waste at 425 °C and 450 °C.

The yield of the oil phase obtained after SCW degradation of PP waste was generally high at all conditions investigated (Figure 3) and was in the range from 73% to 95%. At a temperature of 425 °C and 15 min, only 8–9% of non-degraded PP waste remained in the post-reaction mixture, and the yield of the oil phase for both samples (colored and colorless) was between 85–86%. By increasing the reaction time at 425 °C the yield first increases to 90% at 30 min and afterwards, it starts to decrease. At 450 °C generally slightly higher oil yield was obtained at a specific reaction time as at 425 °C and it decreases with reaction time. The reason for this is that the components of the oil phase are converted into gaseous products and water-soluble components. 

The highest yield of the oil phase was determined at 450 °C after 15 min, and it was 95% for colorless PP and 92% for colored PP. It is obvious that higher temperatures caused faster degradation of PP plastic in SCW to small molecules. As shown in Figure 3, the yields of oil phases from colored PP wastes were generally 1–3% lower than from colorless PP, which is probably due to added colorants in colored plastics, as these can account for up to 10% in the manufacture of plastic products [40].

The results obtained for the oil phase are consistent with the results reported in the literature for degradation of virgin PP in SCW [30], where the maximal yield of the oil phase was achieved at 450 °C and after 1 h of reaction time and it was 91%. The difference between reaction times is a consequence of different heating rates of the reaction mixture to the desired temperature.

**Figure 4:**
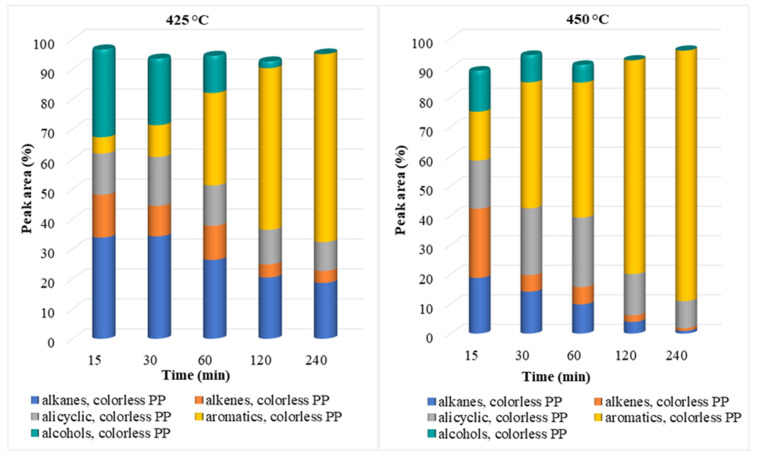
Chemical composition of oil phase after SCW treatment of colorless PP waste at 425 °C and 450 °C for different reaction times.

The chemical composition of the oil phase (according to the peak area (%)), determined using the GC-MS method, was dependent on the reaction conditions and consisted generally of five main groups: saturated and unsaturated aliphatic hydrocarbons, alicyclic hydrocarbons, aromatic hydrocarbons, and alcohols (Figure 4 and Figure S1, Tables S1 and S2 from Supplementary Materials). Furthermore, some amides, ketones, aldehydes, and organic acids were detected in the oil phase. Figure 4 shows the chemical compositions of the oil phase after the degradation of colorless PP waste in SCW. As shown in Figure 4, the highest content of alkanes (33%) and alcohols (29%) was observed in the oil phase obtained at 425 °C and shorter reaction times, and they represented more than 60% of the oil. At these conditions, the PP molecule was degraded to long-chain alkanes (2-methylocracosan, 2-methyltetracosane, and hexacosane), while alcohols were most likely formed by a hydration reaction, where alkenes are hydrogenated to alcohols (e.g., undecane to undecanol). The most represented alcohols detected in the oil phases were undecanol and 1-tridecanol. The third most represented hydrocarbon group in the oil phase at 425 °C were alkenes. In general, the concentration of alkenes decreased from 14 to 3% with the extension of the reaction time up to 240 min, most likely due to hydrogenation, cyclization, aromatization and gasification processes [30]. In contrast, the concentration of aromatic hydrocarbons sharply increased from 5 to 64%. Besides this, the concentration of alicyclic compounds ranged between 13 and 9%.

Oppositely, in the oil obtained at a temperature of 450 °C aromatic compounds were the most represented components when the reaction time was between 30 and 240 min (Figure S1 in the Supplementary Materials). The concentration of aromatic compounds was the lowest at 15 min (17%) and increased sharply with prolongation of time and reached the highest concentration at 240 min, where it was 85% in the case of colorless and 89% in the case of colored PP plastic. Differences in the content of the aromatic compounds in oil phases from colored and colorless plastics were only from 3 to 6%. Among the aromatic compounds, mesitylene and o-xylene predominated, however, at longer reaction times, polycyclic aromatic hydrocarbons (indene, fluorene, anthracene) started to form with the polymerization reaction [41].

#### 3.2.2. Gas Phase

**Figure 5:**
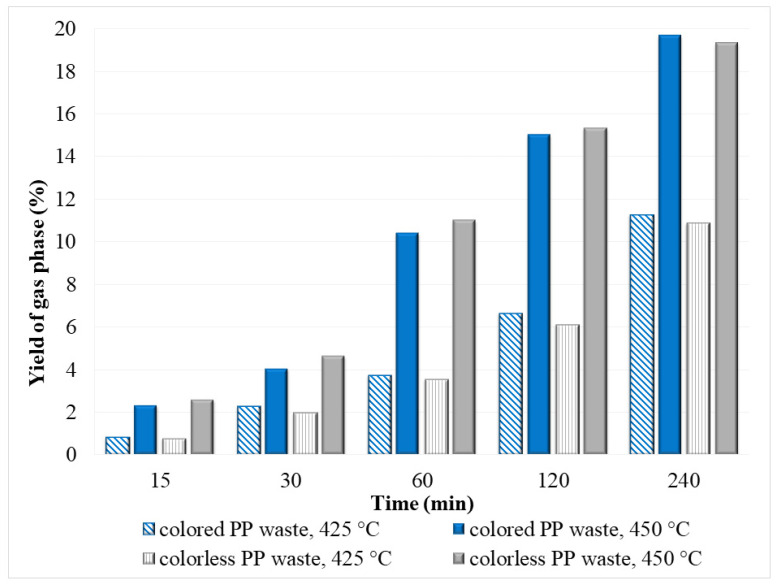
The gas yield obtained after hydrothermal degradation of various PP waste in SCW.

As shown in Figure 5, the yield of the gas phase increased with increasing temperature (from 425 to 450 °C) and reaction time (15–240 min), while the yield of the oil phase decreased (Figure 3). The reason for this was the further decomposition of long-chain alkanes, alkenes, and other products of the oil phase into smaller molecules, resulting in a higher gas yield.

It can also be seen from Figure 5 that there is not much difference in gas yield between waste plastic samples. The lowest gas yield (0.79%) was found after the degradation of colorless PP waste at a reaction time of 15 min and 425 °C, while the highest gas yield (20%) was observed in the case of degradation of colored PP waste at temperature 450 °C and reaction time of 240 min.

**Figure 6:**
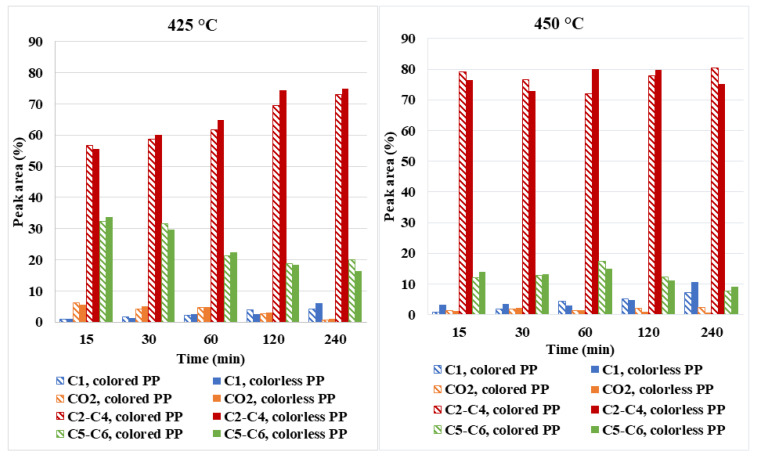
The chemical composition of the gas phase from colorless and colored PP waste.

Figure 6 and Figure S2, Tables S3 and S4 from Supplementary Materials present the chemical composition (according to the peak area (%)) of the gas phase obtained at different reaction conditions and it can be seen that it is mainly composed of light hydrocarbons (C_1_-C_6_) and small amounts of CO_2_. As shown in Figure 6, the methane content increased with increasing reaction time and temperature and reached its maximum concentration (10%) at 450 °C and 240 min in the case of colorless PP plastic (Figure S2 in the Supplementary Materials). The increase of methane concentration in the gas mixture is a consequence of further decomposition of higher gaseous alkanes and alkenes. Despite the fact that PP does not have an oxygen atom in its structure, CO_2_ was detected in the gas phase because the water was involved in the reaction during the decomposition of PP as a reactant (a steam reforming reaction). The highest CO_2_ content in gas mixtures was found at 425 °C and 15 min (for colored PP waste was 6.22%; for colorless PP waste was 5.68%) and decreased with a further extension of the reaction time and reached the lowest value at 240 min and 450 °C in the case of colorless PP waste (0.71%).

Light hydrocarbons C_2_-C_4_ are the most represented components (more than 55%) in all gas mixtures formed after the decomposition of colored and colorless PP wastes. At the temperature of 425 °C, the content of light hydrocarbons C_2_-C_4_ increased with increasing reaction time, while the proportion of hydrocarbons between C_5_ and C_6_ decreased. The most commonly represented component in the gas phase was propane, and it was in the concentration range of 10–26% at 425 °C and between 20–32% at 450 °C. Propane is formed by an endothermic reaction due to the decomposition of oily components. It can be confirmed that long reaction times and high temperatures caused a significant increase in propane concentration due to the hydrogenation of propene (as a result of which its concentration decreased at 450 °C from 18.28 to 1.87%).

Similarly, with rising the temperature from 425 °C to 450 °C and increasing the reaction time up to 240 min, the concentrations of ethane (1.54–24.69%) and isobutane (6.41–24.04%) also increased, while the concentrations of C_5_-C_6_ hydrocarbons decreased as they were cleaved into shorter hydrocarbons (as a result of which their concentration decreased from 33.82 to 16.42% at 425 °C and from 13.83 to 9.09 at 450 °C). Generally, the proportion of unsaturated aliphatic hydrocarbons in the gas mixture was much lower than that of saturated aliphatic hydrocarbons and it decreased with increasing temperature and reaction time from 36% to 6%. The concentrations of individual hydrocarbons (C_1_, C_2_-C_4_, and C_5_-C_6_) and CO_2_ in the gas mixture differ by a maximum of 8% depending on the type of PP waste plastic (colored PP and colorless PP). This finding can be attributed to various additives in waste plastics.

#### 3.2.3. Aqueous Phase

**Figure 7:**
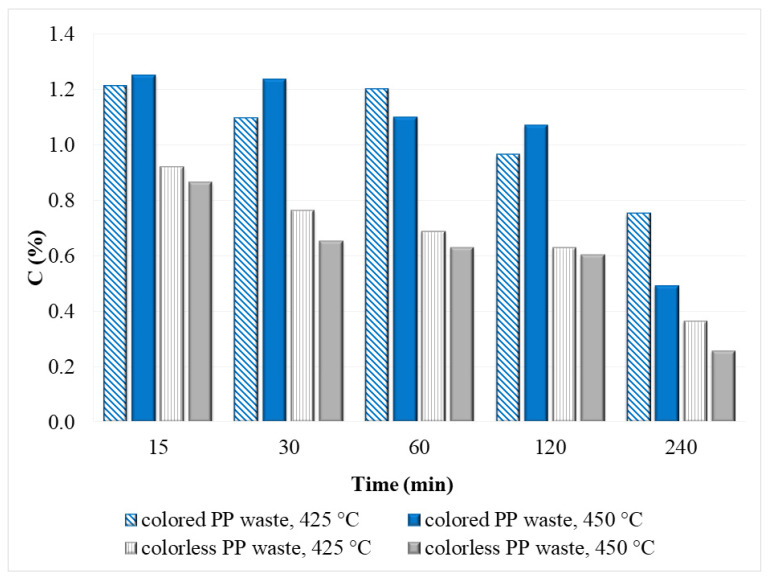
Mass fraction of carbon in the aqueous phase after hydrolysis of PP waste in SCW at 425 and 450 °C.

Figure 7 shows the mass fraction of carbon (%) in the aqueous phase after degradation of colored and colorless PP waste at temperatures of 425 and 450 °C and reaction time from 15 to 240 min. Some carbon from the PP molecule was most likely transferred to the aqueous phase during the degradation of plastic waste. As expected, in the case of colored PP, the mass fraction of carbon in the aqueous phase was higher than in the case of colorless PP plastic. Namely, colored plastics can contain from 0.001 to 2.5% *w/w* of organic pigments and from 0.01 to 5% of soluble colorants [17,40], which were most likely decomposed during the degradation of PP waste in SCW and transferred to the aqueous phase. The maximum carbon content in the aqueous phase was reached at a reaction time of 15 min at both temperatures (for colored PP waste: 1.21% at 425 °C, 1.25% at 450 °C and for colorless waste: 0.86% at 425 °C and 0.91% at 450 °C) and it decreased with increasing time. The lowest mass fraction of carbon in the aqueous phase was detected after 240 min at both temperatures (for colorless PP waste 0.26% and for colored PP waste 0.49%). Based on the gas phase results, it can be confirmed that some degradation products in the aqueous phase were converted to the gas phase, and thus, a decrease in the mass fraction of carbon in the aqueous phase occurred. As shown in Figure 7, the mass fraction of carbon in the aqueous phase is slightly higher for the colored PP plastics. Even though some authors [25,30] have investigated the degradation of polyolefin plastics (especially virgin) in SCW, there is still a lack of data in the literature on the composition of the aqueous phase. Chen et al. determined small amounts of oxygen components (hydroxyisobutyric acid) in the aqueous phase after the degradation of virgin PP at 425 °C and 4 h [30]. Moriya and Emoto determined aldehydes, ketones, secondary alcohols, fatty acids, and phenols after degradation of HDPE in SCW at 425 °C and 2 h [25]. Furthermore, in our previous study, it was found that during the degradation of PE waste some organic matter is transferred to the aqueous phase [8]. By comparing these results to the results for PP obtained in this work it can be observed that the mass fraction of carbon in the water phase after PE degradation was slightly lower than in the case of PP waste and ranged from 0.15 to 0.45% [8].

#### 3.2.4. Solid Phase

**Figure 8:**
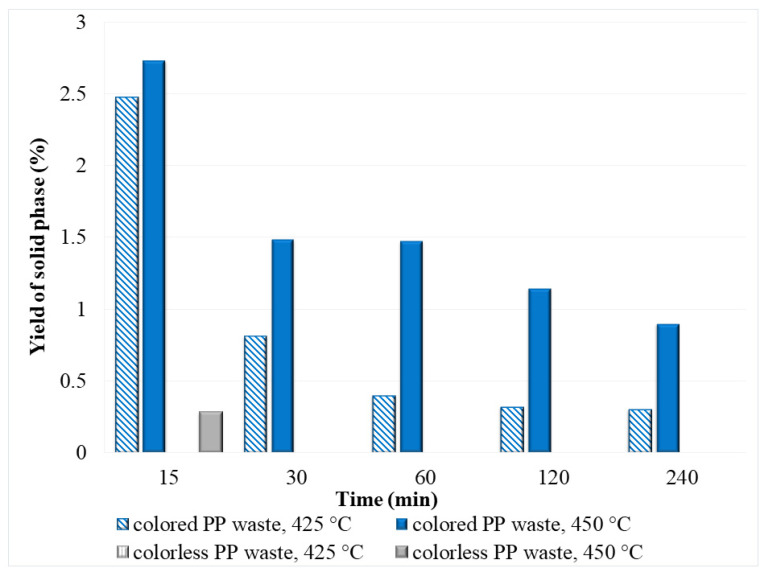
The yield of the solid phase after degradation of colored and colorless PP waste in SCW at 425 and 450 °C.

During the decomposing of PP waste, a black solid residue was formed in the post-reaction mixture (Figure 8). In the case of colored PP waste, the yield of solid residue increased with increasing temperature at constant reaction time and decreased with increasing reaction time at a constant temperature. This is a consequence of the higher reaction rate at higher temperature, so that at 450 °C and short reaction times generally more degradation products (oil phase and solid residue) are formed, while with increasing reaction time at constant temperature these reaction products are further degraded, so that the yield of oil and solid phase decreases, while the yield of gas phase increases. The highest yield of the solid phase for colored PP waste was achieved at 450 °C and 15 min, and it was 2.7%, while the lowest value was reached after 240 min at 425 °C (0.3%). Oppositely, when colorless PP plastic was degraded, char was detected in the reaction mixture only at 450 °C after 15 min (0.28%), while in other experiments, no black residue was determined in the reaction mixture. Compared to colored waste plastics, colorless plastics do not contain dyes that would disintegrate during decomposition. Similarly, Jin et al. reported that after the degradation of various PE wastes (shopping bags, milk jugs, grocery bags) in SCW, different amounts of solid residue (2–20%) are produced. These solid residues after SCW decomposition were attributed to inorganic additives in the plastic waste [31]. Elemental analysis of the solid phase obtained from the colored PP waste after degradation at 450 °C and 120 min showed that the residue consisted of 53.35% C, 3.41% H, 1.21% N, and 0.98% S, while the elemental composition of obtained residue after SCW degradation at 450 °C and 240 min was 51.24% C, 2.55% H, 0.77% N, and 0.56% S. It was found that the results obtained for PP were similar to the elemental composition of solid residue from colored PE waste, which was determined in our previous research (69.35% C, 2.33% H, 1.24% N, and 0.45% S at 450 °C and 120 min, 71.28% C, 3.01% H, 0.15% N, and 0.29% S at 450 °C and 240 min) [8].

From the elemental composition of the solid phases, it can be concluded that the solid phase most likely contains residual hydrocarbons or other organic compounds that are converted to oily or gaseous components by increasing the reaction time in SCW, which is reflected in the lower yield of the solid phase. The remaining solid phase residue most likely contains inorganics (perhaps due to inorganic pigments or other additives in the plastic waste) [31].

### 3.3. Comparison of PP and PE Waste Degradation in SCW

The results of the present work obtained for PP were compared to the results obtained in our previous work, where degradation of PE in SCW was studied under the same conditions [8] and the degradation mechanisms of polyolefins waste in SCW has been proposed. The comparison of the resulting products from waste polyolefins greatly contributes to understanding the chemical recycling of waste polyolefins in SCW.

In the case of PP plastic waste, faster decomposition in SCW is observed compared to PE material. After the degradation of PP waste at a temperature of 425 °C and 15 min, the yield of the oil phase was relatively high, and it was between 85–86%. Besides this, only 8–9% of undegraded PP waste remained in the reaction mixture. In the case of PE waste, the yield of the oil phase obtained at the same conditions was only 29%, and more than 70% of PE plastic was still undegraded. For virgin PP it was observed before that its degradation started already at 380 °C and 120 min of reaction time, where minimal conversion to the oil phase was obtained [30]. On the other hand, colored PE waste in SCW started to decompose at higher temperatures (425 °C) and shorter reaction times (15 min) [8]. The differences in the reaction rates and the composition of the oil and gas phases are obviously due to the different molecular structures of PP and PE. PP tends to disintegrate faster because PP has two types of bonds: C–CH_3_ side bond with lower bond energy (335 kJ/mol) and -CH_2_-CH_2_- bond in the main chain, with bond energy of 348 kJ/mol [30], while PE molecule contains only -CH_2_-CH_2_- bond and consequently more energy (higher temperature) is required for depolymerization. During the decomposition of colored polyolefins, a small amount of solid residue (up to 3.5%) was formed in the reaction mixture, which was attributed to additives (dyes).

**Figure 9:**
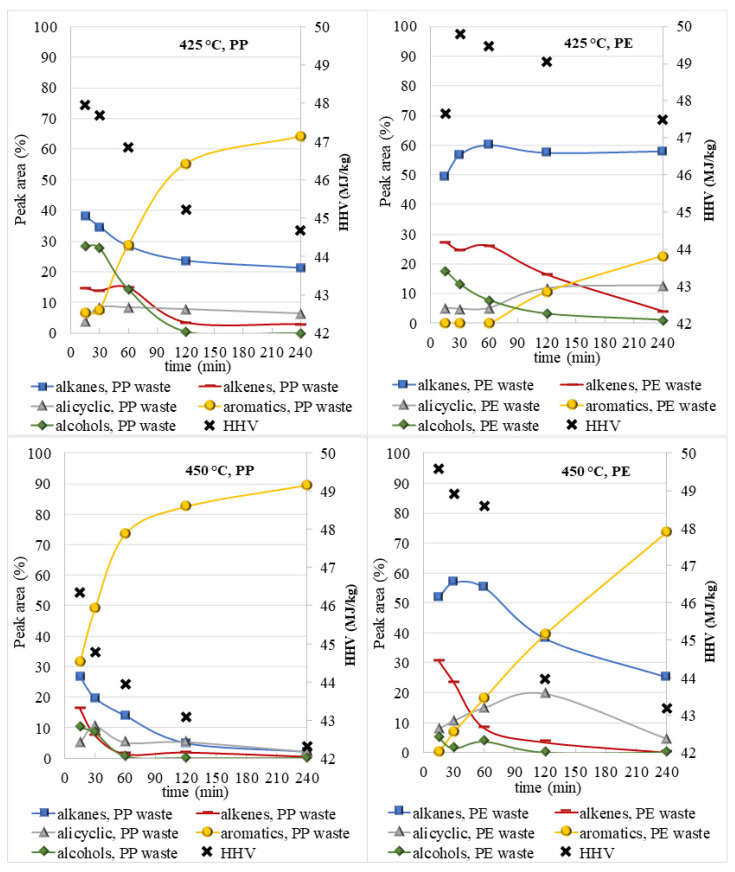
The chemical compositions of oil phases after SCW degradation of colored PE and PP waste and their theoretical HHV values.

In Figure 9, the chemical composition and HHV value of the oil phases from PP and PE are compared. In the case of PE waste, alkanes predominate in the oil phase, as breaking C-C bonds, and cleavage of hydrogen from the main chain accelerated the production of alkanes. The concentration of alkanes in the oil phase ranges between 50 and 60% at 425 °C and between 57 and 26% at 450 °C. In contrast, for PP waste alkane concentrations in the oil phase are significantly lower and decrease with increasing temperature and reaction time from 38% to 21% at 425 °C and from 27% to 0% at 450 °C. Due to the faster decomposition of PP waste, the concentration of alkenes in the oil phases from PP waste (from 15% to 3% at 425 °C and from 16% to 0% at 450 °C) is lower than in the oil phase from PE (from 27% to 4% at 425 °C and from 31% to 0% at 450 °C). With further increase in temperature and prolongated time, alkenes from PP and PE wastes decomposed into other components (alcohols, cycloalkanes, and aromatics). In the case of PP waste, higher alcohol concentrations were found in the reaction mixture than in the case of PE waste. As can be seen from the results, the maximum alcohols content from both plastic wastes were reached at 425 °C and 15 min, and it was almost 30% from PP plastic and only 18% from PE plastic.

Aromatic hydrocarbons from PE plastics appeared only after longer reaction times at both temperatures and the highest content (74%) was achieved at 450 °C and 240 min. In contrast, in the case of PP waste higher concentrations of aromatic hydrocarbons were observed already in shorter reaction times (15 min) and increased with increasing reaction time from 7 to 64% at 425 °C and from 31 to 89% at 450 °C. The most represented aromatic hydrocarbons were mesitylene (PP waste) and o-xylene (PE waste).

Based on the chemical composition of the oil fractions obtained from polyolefins HHV was further evaluated. Crude oil is mainly composed of three types of hydrocarbon components, namely: paraffins, naphthenes, and aromatics. Paraffins are the most common hydrocarbons in crude oil, while naphthenes are important in liquid refineries. Aromatic hydrocarbons generally represent only a small percentage in crude oil, where benzene is the most common substance [42]. Typical HHV values for diesel, petrol/gasoline, crude oil, and fuel oil are 45.60 ± 0.49 MJ/kg, 46.94 ± 0.70 MJ/kg, 45.30 ± 1.47 MJ/kg, and 43.26 ± 1.00 MJ/kg, respectively [43,44]. HHVs, which are estimated from the chemical composition of the oil phases from polyolefins, are very similar to the mentioned fuels. HHVs of oil phases were higher in the case of PE waste and were in the range between 49.8 and 47.5 MJ/kg at 425 °C and between 49.6 and 43.2 MJ/kg at 450 °C. For the oil phases obtained from PP waste, the HHVs decreased with increasing reaction time and were at 425 °C in the range between 48–44.7 MJ/kg and at 450 °C in the range between 46 and 42 MJ/kg. Similar HHVs were determined by Chen et al. for the oil phases from virgin PP after SCW degradation and ranged between 46.3–49.3 MJ/kg [30]. It can be concluded that HHVs from the obtained oil phases after the decomposition of polyolefins in SCW are in the range of petroleum fuels and consequently represent an excellent alternative to fossil fuels.

**Figure 10:**
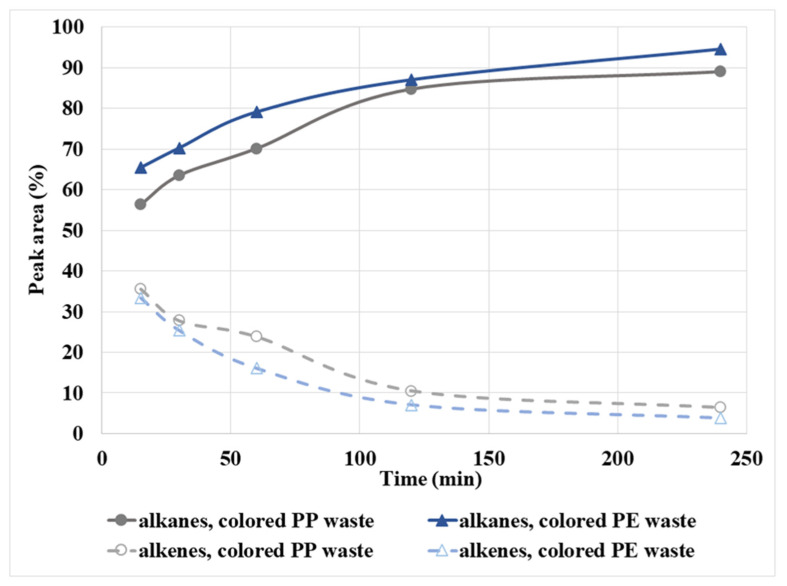
The yield of total alkanes and alkenes in the gas phase after decomposition of colored PE and PP wastes at 450 °C in SCW at different reaction times.

The composition of the gas phases after the degradation of polyolefin wastes is compared in Figure 10. The total yield of the gas phase in both cases of polyolefin plastic increased with temperature and reaction time. The maximum yield of the gas phase was achieved at 450 °C and 240 min and it was 20% for PP plastic and 26% for PE plastic. The gas phases consisted mainly of light hydrocarbons, where hydrocarbons between C_2_ and C_4_ predominated. In general, the proportion of unsaturated aliphatic hydrocarbons in the gas mixture was much lower than the proportion of saturated aliphatic hydrocarbons. At 450 °C it decreased with increasing reaction time up to 240 min from 34% to 3% in the case of colored PE waste and from 36% to 6% in the case of colored PP waste (Figure 10). The most commonly represented component in the gas phases of polyolefins was propane, which was formed by the decomposition of wax by the endothermic reaction and hydrogenation of propene.

#### Degradation Mechanism of Polyolefins

**Figure 11:**
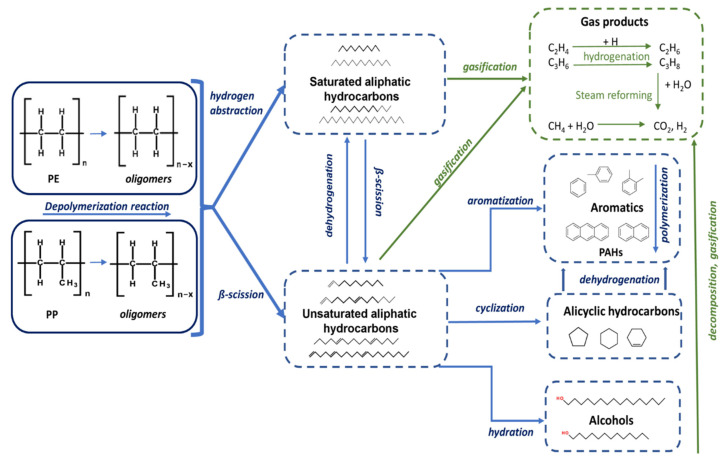
Degradation mechanisms of PP and PE waste in SCW.

Based on the results and analysis, the degradation mechanisms of polyolefins (PP and PE) waste in SCW have been proposed (Figure 11). The composition of the oil phase was dependent on the reaction conditions, but the components formed were very similar for both materials (PE or PP). The degradation pathways for polyolefins are similar, except that the initial degradation of PP occurs at a lower temperature (380–400 °C) and short reaction times (30–60 min) [30] than in the case of PE (425 °C and 15 min) [8] due to differences in the molecular structure of individual plastics. The degradation of PP and PE starts with free radical dissociation, where C-C bonds cleaved into long-chain alkanes. At the same time, α-olefins were formed by the β-scission reaction, while short-chain paraffins were formed by the hydrogen abstraction [8,27,30]. Simultaneously, α-olefins were hydrated to alcohols by the hydration reaction (1-pentadecene to n-pentadecanol) [8,25,45]. In the following, as temperature and time further increased, α-olefins were converted to alicyclic hydrocarbons via cyclization, while a small proportion of alkenes were most likely converted to alkanes by hydrogenation. Subsequently, alicyclic unsaturated hydrocarbons dehydrated to aromatic hydrocarbons over longer reaction times [27,41], while even more extreme conditions led to the formation of polycyclic aromatic hydrocarbons by polymerization of aromatics [41]. At the same time, further cracking of alkanes and alkenes by the reaction of gasification and degradation of other oil components led to an increase in the yield of gaseous products.

Further, the gaseous alkenes at high temperature and longer reaction times decompose to gaseous alkanes by hydrogenation reaction. The results of the GC-MS analyses showed high concentrations of ethane, propane, and butane in the gas mixtures. Further decomposition of the gaseous products increased the methane concentration in the gas samples. Gaseous alkanes can further react with SCW to form CO_2_ and H_2_ by a steam reforming reaction [28,41].

## 4. Conclusions

Temperature and reaction time are important process parameters influencing the yield and the composition of the products obtained by hydrothermal degradation of PP. The resulting products were separated into the gas, oil, water-soluble, and solid phase, among which the oil phase was the main product, while a small amount of solid phase was formed mainly during the decomposition of colored PP and was attributed to the additives contained in the waste plastic. The yields of the oil and gas phases were very similar for both types of PP waste (colored and colorless). The maximum yield of the oil phase was reached at 450 °C and a reaction time of 15 min, and it was 95% in the case of colorless PP and 92% in the case of colored PP. By extending the reaction time at 450 °C to 240 min, the maximum yield of the gas phase (20% for colorless PP and 19% for colored PP) was achieved. By GC-MS analyses, the chemical composition of the gas and oil phases were determined. The oil phase consisted of paraffins, olefins, cyclic and aromatic hydrocarbons, and alcohols. In the gas phase light hydrocarbons such as methane, ethene, ethane, propane, propene, 1-butene, butane, 1-pentene, pentane, 1-hexene, hexane, and a small amount of CO_2_ were present, where propane was the most commonly represented component.

The results obtained for hydrothermal degradation of PP waste were compared with the results obtained for hydrothermal degradation of PE waste from our previous study and the decomposition mechanism of polyolefins waste in SCW was presented. The difference in molecular structure between the polymers leads to a slightly faster degradation in the case of PP waste. The important finding is that similar degradation products are obtained from both polyolefins.

The HHVs of the oil phases from PP and PE, estimated from the chemical composition of the oil, ranged between 48 and 42 MJ/kg in the case of PP plastics and between 49.8 and 43.2 MJ/kg in the case of PE plastics. The HHVs of the oil phases from polyolefins plastic wastes were in the range of fossil fuels (diesel, gasoline, crude oil).

The presence of colorants or additives in the plastic waste has a little effect on the yield of the degradation products and compounds formed, however there are some minor differences in the fractions of individual compounds in the oil and gas phases from colorless and colored PP waste. Nevertheless, it can be concluded that the chemical components obtained from polyolefins waste (PE in PP) have a great potential in the production of fuels (gases and oils) or as platform chemicals.

## Figures and Tables

**Table 1:** Elemental analysis and thermal properties of PP waste.

	Black PP Waste	Blue PP Waste	Colorless PP Waste
C (wt %)	83.44	81.87	84.37
H (wt %)	13.78	13.41	13.32
N (wt %)	0.24	0.45	0.19
S (wt %)	1.05	1.30	0.98
O* (wt %)	1.49	2.97	1.14
*T*_cr_ (°C)	123.04	122.33	119.36
Δ*H*_c_ (J/g)	75.76	75.21	74.64
*T*_m_ (°C)	168.87	167.99	169.90
Δ*H*_m_ (J/g)	79.49	78.25	72.92

## Data Availability

Not applicable.

## Supplementary Materials

The following supporting information can be downloaded at: https://www.mdpi.com/article/10.3390/polym14204415/s1, Figure S1: GCMS chromatogram of the oil phase after hydrothermal degradation of colored PP waste at 450°C and 30 min. Figure S2: GC-MS chromatogram of gas phase after hydrothermal degradation of colorless PP waste at 450 °C and 240 min. Table S1: Hydrocarbon groups and alcohols and their most represented components (peak area (%)) in oil phase after decomposition of colored and colorless PP waste in SCW at 425 °C. Table S2: Hydrocarbon groups and alcohols and their most represented components (peak area (%)) in oil phase after decomposition of colored and colorless PP waste in SCW at 450 °C. Table S3: Chemical composition of the gas phase (peak area (%)) after hydrothermal decomposition of colored and colorless PP waste at 425 °C. Table S4: Chemical composition of the gas phase (peak area (%)) after hydrothermal decomposition of colored and colorless PP waste at 450 °C.
