# Supplementary material for: Chemical Recycling of Polyolefins Waste Materials Using Supercritical Water
Source: Polymers (Basel). 2022 Oct 19;14(20):4415. doi: 10.3390/polym14204415 (PMC9609547; doi:10.3390/polym14204415)
Supplement: Supplementary file 1 [file polymers-14-04415-s001.zip › polymers-1940723-supplementary.pdf]

Figure S1: GCMS chromatogram of the oil phase after hydrothermal degradation of colored PP waste at 450°C and 30 min.

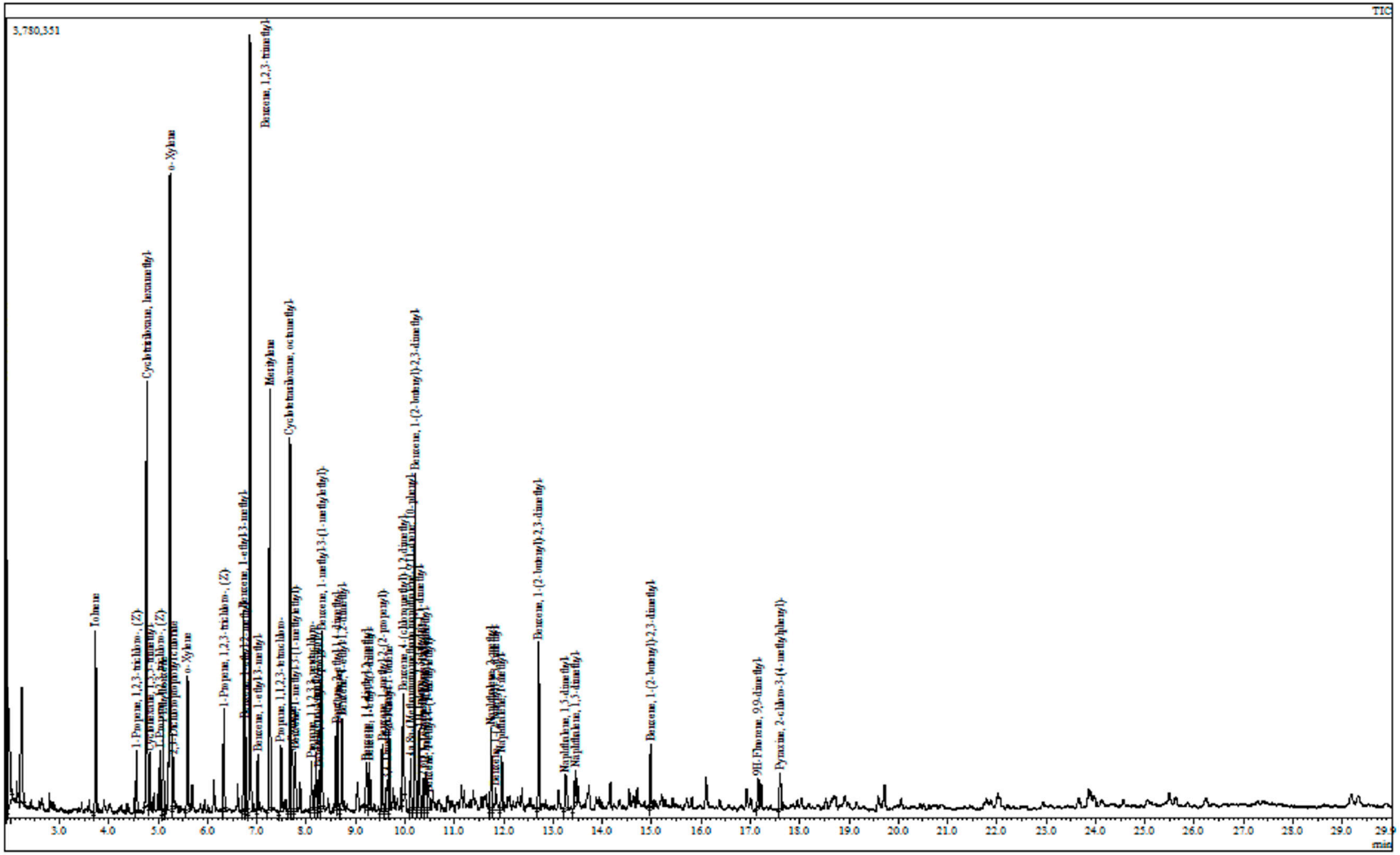

Table S1: Hydrocarbon groups and alcohols and their most represented components (peak area (%)) in oil phase after decomposition of colored and colorless PP waste in SCW at 425 °C.

| **Compound name /reaction conditions** | **15 min, 425 °C** | | **30 min, 425 °C** | | **60 min, 425 °C** | | **120 min, 425 °C** | | **240 min, 425 °C** | |
|---|---|---|---|---|---|---|---|---|---|---|
| | **Colored PP waste** | **Colorless PP waste** | **Colored PP waste** | **Colorless PP waste** | **Colored PP waste** | **Colorless PP waste** | **Colored PP waste** | **Colorless PP waste** | **Colored PP waste** | **Colorless PP waste** |
| | Peak area (%) | | Peak area (%) | | Peak area (%) | | Peak area (%) | | Peak area (%) | |
| **Saturated Aliphatic Hydrocarbons (SUM)** | **38.26** | **33.79** | **34.83** | **34.19** | **28.45** | **26.31** | **23.70** | **20.45** | **21.28** | **18.63** |
| 2,5,5-trimethylheptane | 0.59 | 5.76 | 6.09 | 6.02 | 5.41 | 6.33 | 4.91 | 4.15 | 3.35 | 3.22 |
| 4-methylheptane | 1.59 | 2.53 | 0.69 | 1.88 | 2.48 | 2.19 | 2.72 | 3.01 | 1.81 | 2.15 |
| 2,6,6-trimethyloctane | 2.34 | 2.42 | nd | nd | 1.35 | nd | 1.50 | nd | nd | nd |
| Undecane | nd | 3.41 | 1.36 | 2.99 | nd | 1.01 | 1.38 | 1.44 | 1.02 | 1.04 |
| 4,6-dimethylundecane | 2.31 | 1.38 | nd | nd | nd | nd | nd | nd | nd | nd |
| 4,6-dimethyldodecane | 2.58 | 6.49 | 3.43 | 2.16 | 2.73 | 3.33 | 2.06 | 2.45 | 3.10 | 2.68 |
| 2,6,10-trimethyldodecane | nd | nd | nd | nd | 2.02 | nd | 0.72 | nd | 1.15 | nd |
| Tetradecane | 5.62 | 1.64 | 1.94 | 2.55 | 0.14 | 1.79 | 0.99 | 0.78 | nd | 0.56 |
| Pentadecane | nd | nd | nd | nd | nd | nd | 2.61 | 1.16 | 0.99 | 0.89 |
| Hexacosane | 5.81 | 1.37 | 7.92 | 8.23 | nd | 3.58 | nd | 0.78 | 0.55 | 0.63 |
| heptadecane | nd | nd | 2.94 | 4.55 | nd | 2.64 | 0.98 | 1.18 | nd | nd |
| Heneicosane | 5.12 | 3.18 | 3.22 | 2.86 | 2.78 | 1.67 | 1.36 | nd | 1.09 | nd |
| 2-methyltetracosane | 7.3 | 4.01 | 4.04 | 2.01 | 1.01 | 0.35 | nd | nd | nd | nd |
| 2-methyloctacosane | 5.0 | nd | 3.20 | nd | nd | nd | nd | nd | nd | nd |
| **Unsaturated Aliphatic Hydrocarbons (SUM)** | **14.75** | **14.26** | **13.87** | **10.18** | **14.92** | **11.41** | **3.39** | **4.36** | **2.81** | **3.99** |
| 4,5-dimethylhexene | 3.55 | 2.87 | 1.69 | 1.78 | 2.28 | 1.41 | 0.78 | 1.01 | 0.65 | 0.92 |
| 2,4-dimethyl-1-decene | 4.12 | 3.35 | 0.74 | 3.59 | 2.07 | 2.63 | nd | 1.44 | nd | nd |
| 2,4-dimethyl-2-decene | 1.46 | 0.85 | 4.09 | 2.44 | 2.30 | 1.96 | nd | 0.99 | nd | nd |
| 2,2-dimethyl-3-decene | nd | nd | nd | nd | 4.60 | 4.20 | 2.61 | 0.78 | 0.64 | nd |
| 2,3,4-trimethyl-1-Tetradecene | nd | 3.18 | nd | nd | nd | nd | nd | nd | nd | nd |
| 1-tetradecene | 1.98 | 0.92 | nd | 0.55 | nd | nd | nd | nd | nd | nd |
| 1-hexacosene | 3.64 | nd | 0.77 | nd | nd | nd | nd | nd | nd | nd |
| methyl-1-undecene | nd | 0.85 | 0.44 | nd | nd | nd | nd | nd | nd | nd |

| | | | | | | | | | | |
|---|---|---|---|---|---|---|---|---|---|---|
| **Alicyclic Hydrocarbons (SUM)** | **3.97** | **13.78** | **8.45** | **16.32** | **8.46** | **13.45** | **7.85** | **11.56** | **6.41** | **9.63** |
| cyclopropane derivates | 0.99 | 1.87 | 0.88 | 1.36 | 0.39 | nd | nd | nd | nd | 1.21 |
| cyclopentane derivates | nd | nd | 0.50 | 2.12 | nd | 1.24 | 0.6 | 1.21 | 0.37 | nd |
| cyclopentene derivates | 0.93 | nd | 0.63 | 0.33 | nd | 1.15 | nd | 0.77 | nd | 0.88 |
| cyclohexane derivates | 2.04 | 10.20 | 6.43 | 8.58 | 8.07 | 8.14 | 7.25 | 7.99 | 6.04 | 5.23 |
| cyclohexene derivates | nd | 1.70 | nd | 0.56 | nd | nd | nd | nd | nd | nd |
| **Aromatics Hydrocarbons (SUM)** | **6.59** | **5.45** | **7.52** | **10.63** | **28.79** | **30.87** | **55.38** | **53.96** | **64.34** | **62.79** |
| naphthalene derivates | nd | nd | nd | nd | 1.10 | 1.89 | 1.92 | 2.56 | 5.00 | 3.89 |
| benzene derivates | 6.59 | 5.45 | 7.52 | 10.63 | 27.69 | 28.98 | 53.46 | 51.40 | 59.34 | 58.90 |
| **Alcohols (SUM)** | **28.41** | **29.20** | **27.85** | **22.13** | **14.35** | **12.31** | **0.51** | **2.15** | / | / |
| 1-butanol | 1.48 | nd | nd | nd | nd | nd | nd | nd | nd | nd |
| 4,4-dimethyl-3-hexanol | nd | nd | nd | 2.31 | 2.10 | 3.56 | 0.27 | 1.58 | nd | nd |
| 2,4-dimethyl-1-heptanol | 0.60 | 2.92 | 4.43 | 3.15 | nd | nd | nd | nd | nd | nd |
| 2-hexyl-1-dodecanol | 1.41 | 2.62 | nd | 2.01 | nd | 1.02 | nd | 0.51 | nd | nd |
| 1-tridecanol | 4.06 | 2.03 | 5.13 | 5.69 | 1.90 | nd | nd | nd | nd | nd |
| undecanol | 10.21 | 5.97 | 10.3 | 8.54 | 4.85 | 5.69 | nd | nd | nd | nd |
| 4,4-dimethyl-3-dodecanol | nd | 2.53 | nd | nd | nd | nd | nd | nd | nd | nd |
| **Others (SUM)** | **8.02** | **3.53** | **7.48** | **6.58** | **5.03** | **5.65** | **9.17** | **7.52** | **5.16** | **4.96** |

**nd: not detected**

Table S2: Hydrocarbon groups and alcohols and their most represented components (peak area (%)) in oil phase after decomposition of colored and colorless PP waste in SCW at 450 °C.

| **Compound name /reaction conditions** | **15 min, 450 °C** | | **30 min, 450 °C** | | **60 min, 450 °C** | | **120 min, 450 °C** | | **240 min, 450 °C** | |
|---|---|---|---|---|---|---|---|---|---|---|
| | **Colored PP waste** | **Colorless PP waste** | **Colored PP waste** | **Colorless PP waste** | **Colored PP waste** | **Colorless PP waste** | **Colored PP waste** | **Colorless PP waste** | **Colored PP waste** | **Colorless PP waste** |
| | Peak area (%) | | Peak area (%) | | Peak area (%) | | Peak area (%) | | Peak area (%) | |
| **Saturated Aliphatic Hydrocarbons (SUM)** | **26.61** | **18.87** | **19.60** | **14.19** | **13.78** | **9.87** | **4.84** | **3.95** | **1.97** | **0.95** |
| 2,4-dimethylheptane | nd | nd | 1.59 | 1.14 | nd | 1.75 | nd | 0.27 | nd | 0.13 |
| 2,5,5-trimethylheptane | nd | 2.39 | nd | 2.01 | nd | 2.25 | 0.23 | nd | nd | nd |
| 4-methylheptane | 3.46 | 0.69 | 2.14 | 1.11 | 1.74 | 2.39 | 0.86 | 0.69 | 0.31 | 0.14 |
| 2,6,6-trimethyloctane | 0.57 | nd | nd | nd | nd | nd | nd | nd | nd | nd |
| Undecane | 2.53 | 0.91 | 1.72 | nd | 0.73 | 0.31 | 0.86 | 0.34 | 0.28 | 0.22 |
| Dodecane | nd | 1.14 | 1.19 | nd | 0.87 | nd | 0.48 | 0.55 | 0.39 | nd |
| 4-methyldodecane | 2.12 | nd | 1.27 | 1.54 | nd | nd | nd | nd | nd | nd |
| 4,6-dimethyldodecane | 3.66 | nd | 1.64 | nd | 1.24 | 0.55 | nd | 0.20 | nd | nd |
| 2,6,10-trimethyldodecane | nd | nd | 1.15 | 0.67 | nd | nd | nd | nd | nd | nd |
| Tetradecane | 3.34 | 0.76 | 1.45 | 0.33 | nd | nd | 0.68 | nd | nd | nd |
| Pentadecane | nd | 1.50 | 0.52 | nd | 0.99 | nd | 0.52 | nd | nd | nd |
| hexadecane | 1.19 | 0.58 | nd | nd | 0.45 | nd | nd | nd | nd | nd |
| heptadecane | nd | nd | 0.56 | 0.35 | nd | nd | nd | nd | nd | nd |
| Oktadecane | nd | nd | 1.21 | nd | nd | 0.55 | nd | nd | nd | nd |
| Heneicosane | 4.98 | 3.19 | 3.14 | nd | nd | nd | nd | nd | nd | nd |
| 2-metiltetracosane | 1.62 | 1.92 | nd | nd | nd | nd | nd | nd | nd | nd |
| **Unsaturated Aliphatic Hydrocarbons (SUM)** | **16.09** | **23.43** | **7.31** | **5.59** | **1.23** | **5.89** | **1.75** | **2.25** | **0.40** | **0.92** |
| 2,4,6-trimethyl-3-heptene | 1.57 | 0.49 | nd | nd | nd | nd | nd | nd | nd | nd |
| 1-octene | nd | 2.79 | nd | 1.93 | nd | nd | nd | nd | nd | nd |
| 4,5-dimethyl-1-hexene | 2.66 | 2.44 | 2.27 | nd | nd | 1.44 | nd | nd | nd | nd |
| 2,4-dimethyl-1-decene | 5.46 | 5.35 | 2.35 | 1.89 | nd | 1.40 | nd | 1.01 | nd | nd |
| 2,4-dimethyl-2-decene | 4.81 | 1.29 | nd | nd | nd | nd | nd | nd | nd | nd |
| 2,2-dimethyl-3-decene | nd | 1.94 | 1.12 | 0.69 | 0.82 | 2.35 | 0.55 | 1.03 | 0.24 | 0.65 |

| | | | | | | | | | | |
|---|---|---|---|---|---|---|---|---|---|---|
| **Alicyclic Hydrocarbons (SUM)** | **5.03** | **16.31** | **10.35** | **22.68** | **5.18** | **23.56** | **5.31** | **13.94** | **2.14** | **9.11** |
| cyclopropane derivates | nd | 1.03 | nd | nd | nd | nd | nd | nd | nd | nd |
| cyclopentane derivates | nd | 0.36 | nd | 2.21 | 0.23 | 3.27 | 0.6 | 1.23 | nd | 0.43 |
| cyclopentene derivates | nd | 0.76 | 1.57 | nd | nd | nd | nd | nd | nd | nd |
| cyclohexane derivates | 5.03 | 14.16 | 8.31 | 18.17 | 4.68 | 19.97 | 4.71 | 12.32 | 2.14 | 8.68 |
| Cyclooctane derivates | nd | nd | nd | 2.30 | nd | 0.32 | nd | 0.39 | nd | nd |
| **Aromatics Hydrocarbons (SUM)** | **31.35** | **16.58** | **49.00** | **42.69** | **73.39** | **45.75** | **82.43** | **72.48** | **89.44** | **84.93** |
| naphthalene derivates | nd | nd | 3.04 | 0.83 | 4.43 | 1.44 | 11.33 | 10.05 | 12.26 | 11.99 |
| benzene derivates | 31.35 | 16.58 | 45.96 | 41.86 | 68.66 | 44.75 | 67.43 | 60.84 | 73.14 | 70.37 |
| **Alcohols (SUM)** | **10.08** | **13.79** | **8.43** | **9.14** | **0.60** | **5.87** | / | / | / | / |
| 2-hexanol | nd | nd | nd | nd | 0.37 | nd | nd | nd | nd | nd |
| 4,4-dimethyl-3-hexanol | nd | nd | 1.14 | nd | nd | nd | nd | nd | nd | nd |
| n-tetradecanol | 0.93 | nd | nd | nd | nd | nd | nd | nd | nd | nd |
| 2,4-dimethyl-1-heptanol | 1.06 | 0.78 | nd | 4.01 | nd | 0.81 | nd | nd | nd | nd |
| 4-heptanol | nd | 0.57 | nd | 0.41 | nd | 0.81 | nd | nd | nd | nd |
| 4-methyl-5-decanol | nd | 1.72 | nd | 1.02 | nd | 0.97 | nd | nd | nd | nd |
| 2-hexyl-1-dodecanol | 1.28 | nd | nd | nd | nd | nd | nd | nd | nd | nd |
| Docosanol | nd | 1.86 | nd | nd | nd | nd | nd | nd | nd | nd |
| 1-tridecanol | 1.51 | nd | 1.11 | 0.97 | nd | 0.69 | nd | nd | nd | nd |
| 1-undecanol | nd | 9.21 | 5.17 | nd | 0.23 | 2.31 | nd | nd | nd | nd |
| 4,4-dimethyl-3-dodecanol | 2.11 | nd | nd | nd | nd | 0.45 | nd | nd | nd | nd |
| **Others (SUM)** | **10.84** | **11.02** | **5.31** | **5.71** | **5.28** | **9.07** | **5.67** | **7.38** | **6.06** | **4.09** |

**nd: not detected**

Figure S2: GC-MS chromatogram of gas phase after hydrothermal degradation of colorless PP waste at 450 °C and 240 min.

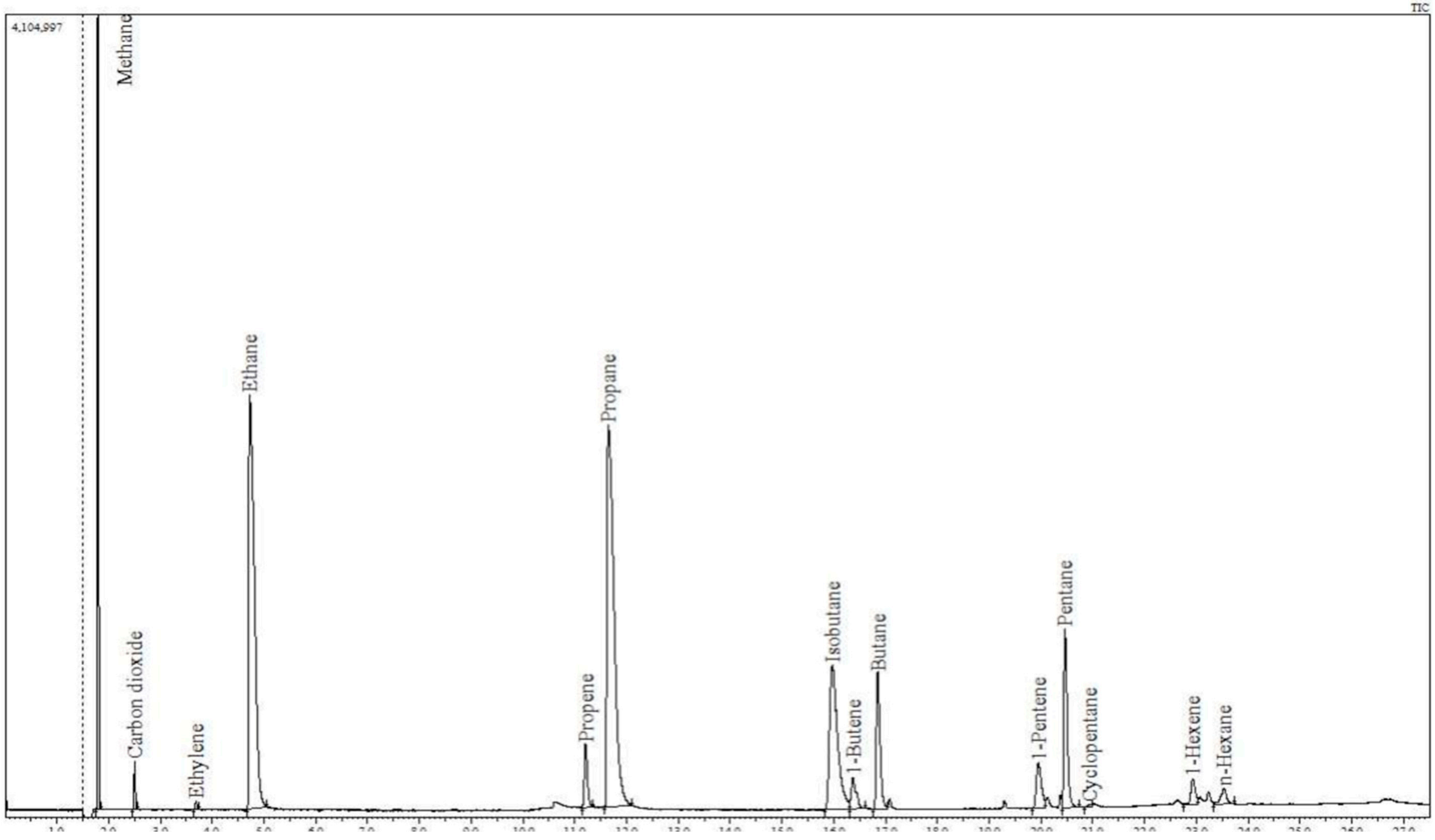

Table S3: Chemical composition of the gas phase (peak area (%)) after hydrothermal decomposition of colored and colorless PP waste at 425 °C.

| **Compound name /reaction conditions** | **15 min, 425 °C** | | **30 min, 425 °C** | | **60 min, 425 °C** | | **120 min, 425 °C** | | **240 min, 425 °C** | |
|---|---|---|---|---|---|---|---|---|---|---|
| | **Colored PP waste** | **Colorless PP waste** | **Colored PP waste** | **Colorless PP waste** | **Colored PP waste** | **Colorless PP waste** | **Colored PP waste** | **Colorless PP waste** | **Colored PP waste** | **Colorless PP waste** |
| | Peak area (%) | | Peak area (%) | | Peak area (%) | | Peak area (%) | | Peak area (%) | |
| **Methane** | 0.99 | 1.02 | 1.65 | 1.23 | 2.12 | 2.45 | 3.84 | 2.55 | 4.18 | 5.96 |
| **$CO_2$** | 6.22 | 5.68 | 4.12 | 5.16 | 4.66 | 4.71 | 2.63 | 3.18 | 0.71 | 1.15 |
| **Ethene** | 2.15 | 1.97 | 2.45 | 2.01 | 3.24 | 3.69 | 1.89 | 0.99 | 0.31 | 0.85 |
| **Ethane** | 1.96 | 1.54 | 3.11 | 2.88 | 3.52 | 3.21 | 4.39 | 5.18 | 5.31 | 4.99 |
| **Propene** | 10.23 | 9.16 | 14.79 | 15.96 | 16.55 | 16.87 | 10.22 | 11.02 | 6.22 | 5.11 |
| **Propane** | 10.34 | 11.15 | 10.86 | 11.99 | 12.47 | 12.85 | 20.28 | 21.68 | 25.87 | 26.44 |
| **Isobutane** | 9.26 | 6.41 | 9.18 | 10.02 | 12.56 | 12.5 | 17.96 | 19.33 | 24.04 | 23.91 |
| **1- Butene** | 10.33 | 12.21 | 6.22 | 5.69 | 5.13 | 6.25 | 4.48 | 4.64 | 4.01 | 5.13 |
| **Butane** | 12.33 | 13.11 | 12.13 | 11.63 | 8.16 | 9.44 | 10.22 | 11.47 | 7.16 | 8.40 |
| **1-Pentene** | 8.15 | 9.16 | 6.05 | 5.32 | 3.66 | 4.01 | 3.68 | 2.15 | 1.99 | 0.75 |
| **Pentane** | 13.91 | 12.69 | 15.11 | 16.01 | 10.99 | 12.15 | 11.54 | 12.15 | 15.12 | 14.49 |
| **1-Hexene** | 4.13 | 4.12 | 5.12 | 4.2 | 3.15 | 2.99 | 1.11 | 2.15 | 2.9 | 1.01 |
| **n-Hexane** | 6.15 | 7.85 | 5.16 | 4.11 | 3.52 | 3.34 | 2.44 | 1.99 | 0.13 | 0.22 |
| C2-C4 | 56.6 | 55.55 | 58.74 | 60.18 | 61.63 | 64.81 | 69.44 | 74.31 | 72.92 | 74.83 |
| **SUM** | 96.15 | 96.07 | 95.95 | 96.21 | 89.73 | 94.46 | 94.68 | 98.48 | 97.95 | 98.41 |

Table S4: Chemical composition of the gas phase (peak area (%)) after hydrothermal decomposition of colored and colorless PP waste at 450 °C.

| Compound name /reaction conditions | 15 min, 450 °C | | 30 min, 450 °C | | 60 min, 450 °C | | 120 min, 450 °C | | 240 min, 450 °C | |
|---|---|---|---|---|---|---|---|---|---|---|
| | **Colored PP waste** | **Colorless PP waste** | **Colored PP waste** | **Colorless PP waste** | **Colored PP waste** | **Colorless PP waste** | **Colored PP waste** | **Colorless PP waste** | **Colored PP waste** | **Colorless PP waste** |
| | Peak area (%) | | Peak area (%) | | Peak area (%) | | Peak area (%) | | Peak area (%) | |
| **Methane** | 0.88 | 3.33 | 1.72 | 3.35 | 4.39 | 2.96 | 5.16 | 4.73 | 7.20 | 10.60 |
| **$CO_2$** | 1.26 | 1.07 | 1.76 | 2.15 | 1.42 | 1.33 | 2.00 | 0.93 | 2.27 | 0.64 |
| **Ethene** | 0.39 | 0.91 | 1.22 | 1.15 | 0.41 | 0.24 | 0.18 | 0.33 | 0.11 | 0.16 |
| **Ethane** | 10.15 | 17.41 | 10.26 | 15.68 | 17.86 | 16.21 | 16.09 | 22.38 | 19.01 | 24.69 |
| **Propene** | 18.28 | 16.24 | 10.72 | 10.15 | 10.43 | 5.19 | 2.91 | 3.79 | 1.87 | 2.01 |
| **Propane** | 21.66 | 20.18 | 24.14 | 19.15 | 25.72 | 28.15 | 29.26 | 31.06 | 32.75 | 28.75 |
| **Isobutane** | 10.08 | 5.68 | 12.33 | 10.35 | 5.21 | 18.97 | 19.16 | 11.53 | 20.14 | 12.35 |
| **1- Butene** | 14.45 | 11.92 | 13.10 | 11.25 | 6.93 | 5.98 | 4.02 | 4.15 | 2.03 | 1.66 |
| **Butane** | 4.01 | 4.02 | 4.88 | 5.22 | 5.51 | 5.26 | 6.16 | 6.44 | 4.53 | 5.46 |
| **1-Pentene** | 0.99 | 1.11 | 0.50 | 1.23 | 2.01 | 2.19 | 1.63 | 0.97 | 1.46 | 0.59 |
| **Pentane** | 9.34 | 9.22 | 9.66 | 8.47 | 10.84 | 9.47 | 7.95 | 8.39 | 5.13 | 6.32 |
| **1-Hexene** | 1.50 | 3.12 | 2.20 | 2.56 | 4.05 | 2.25 | 1.75 | 1.21 | 0.88 | 1.23 |
| **n-Hexane** | 0.16 | 0.38 | 0.50 | 0.88 | 0.59 | 1.14 | 0.99 | 0.50 | 0.31 | 0.95 |
| **C2-C4** | 79.02 | 76.36 | 76.65 | 72.95 | 72.07 | 80.00 | 77.78 | 79.68 | 80.44 | 75.08 |
| **SUM** | 93.15 | 94.59 | 92.99 | 91.59 | 95.37 | 99.34 | 97.26 | 96.41 | 97.69 | 95.41 |
